# Application of Bacteriophages to Control *Vibrio alginolyticus* Contamination in Oyster (*Saccostrea glomerata*) Larvae

**DOI:** 10.3390/antibiotics9070415

**Published:** 2020-07-16

**Authors:** Tuan Son Le, Paul C. Southgate, Wayne O’Connor, Sang V. Vu, D. İpek Kurtböke

**Affiliations:** 1 Research Institute for Marine Fisheries, 224 Le Lai, Ngo Quyen, Hai Phong 180000, Vietnam; letuanson1987@gmail.com or; 2 GeneCology Research Centre and School of Science and Engineering, University of the Sunshine Coast, 90 Sippy Downs Drive, Sippy Downs, Queensland 4556, Australia; vuvansangts50@gmail.com or; 3 Australian Centre for Pacific Islands Research and School of Science and Engineering, University of the Sunshine Coast, Maroochydore, Queensland 4556, Australia; psouthgate@usc.edu.au; 4 NSW Fisheries, Port Stephens Fisheries Institute, Taylors Beach 2316, Australia; wayne.oconnor@dpi.nsw.gov.au

**Keywords:** *Vibrio alginolyticus*, vibriosis, bacteriophage, biocontrol, oyster larvae

## Abstract

Mortalities of bivalve larvae and spat linked with *Vibrio* spp. infection have been described in hatcheries since 1959, causing potential development of resistant bacteria. A reliable and sustainable solution to this problem is yet to be developed. Potential treatment of bacterial infection with bacteriophages is gaining interest in aquaculture as a more sustainable option for managing *Vibrio* spp. infection. This study assessed the effectiveness of bacteriophages (Φ-5, Φ-6, and Φ-7) against pathogenic *Vibrio* isolates (USC-26004 and USC-26005). These phage isolates were found to belong to the *Myoviridae* viral family. A total of 212 ORFs of Φ-5 were identified and annotated. The genome of this phage contained putative thymidine kinase and lysin enzyme. During infections with phages, the OD values of the isolates USC-26005 and USC-26004 remained stable at a much lower reading compared to the control after 9 h of incubation. Mortality rate of oyster (*Saccostrea glomerata*) larvae was 28.2 ± 3.5% in the bacteriophage treatment group, compared to 77.9 ± 9.1% in the bacterial treatment group after 24 h incubation. Findings of this study indicate that lytic phages might be utilized as potential bio-control agents of luminescent bacterial disease in oyster hatcheries.

## 1. Introduction

Global oyster production accounted for 31% of total world mollusk production in 2016 [1] and a significant proportion of this output relied on hatchery production [2]. However, bivalve hatcheries are constantly threatened by bacterial diseases [3] and in most cases, members of the genus *Vibrio* have been identified as significant pathogens in production environments [4]. Since the first description of *Vibrio* species as the causative agents of disease in bivalve larvae [5], numerous studies have identified pathogenic *Vibrio* spp., including *V. tubiashii* [3], *V. splendidus* biovar II [6], and *V. ostreicida* [7] associated with infections that have caused major losses for shellfish farmers [8].

Major activities within oyster hatcheries include broodstock holding, spawning induction, rearing and setting of larvae, and the culture and production of live microalgae as a larval food source [2]. Three key vectors for potential *Vibrio* spp. contamination in these hatcheries include broodstock, microalgae cultures, and seawater supply [9,10].

Antibiotics have been used to treat vibriosis in aquaculture systems [11]; however, there are considerable concerns regarding the long-term use of antibiotics because of the potential for development of antibiotic-resistant bacteria [11], which could be exacerbated by transmission of antibiotic resistance genes into different members of hatchery microbiota by horizontal transfer mechanisms [10]. Karunasagar et al. [12] indicated that mass mortality of black tiger shrimp (*Penaeus monodon*) larvae was caused by multi-drug resistant *V. harveyi* strains with reported resistance to cotrimoxazole, chloramphenicol, erythromycin, and streptomycin. Similarly, the *Vibrio* spp. isolated from fish-pond facilities in Nigeria were resistant to tetracyclines (100%), oxytetracycline (99.4%), and chloramphenicol (73.1%) [13]. Resistance to antibiotics by *Vibrio* spp. has also been reported in infected bivalve mollusks; examples include resistance to ampicillin, vancomycin, and erythromycin by 15 strains of *V. alginolyticus* [14]. In some countries, use of antibiotics in aquaculture species is now prohibited because of potential side effects and development of harmful residues [15].

Although mortalities of bivalve larvae and spat linked with *Vibrio* spp. infections have been described in hatcheries since 1959 [5], a reliable and sustainable solution to disease control is yet to be developed. Treatment of bacterial infections with bacteriophages is gaining considerable interest as a safer alternative for its potential to reduce or remove bacterial contamination in aquaculture systems [11]. Examples include bacteriophage control of *V. parahyemolyticus* infection in adult oysters (*Ostrea plicatula*) [16] and in striped catfish (*Pangasianodon hypophthalmus*) [17], and bacteriophage treatment of *V. coralliilyticus* infection in Pacific oyster (*Crassostrea gigas*) [18].

In addition, phage enzymes can have antibacterial activities. At the end of the lytic cycle of double-stranded DNA bacteriophages, holin, and lysin enzymes are created in order for bacteriophages to exit the infected bacterial host cell. With the help of holins which form large and non-selective pores in the cytoplasmic membrane, lysins can interact with the peptidoglycan substrate and cause cell lysis [19]. The holin and lysin interaction is known as the lambda paradigm [20]. Lysin is considered to be one of the most effective anti-microbial agents because ~50% of bacteria on the planet are killed by the bacteriophage lytic cycle every 2 days [21]. Lysins, which are greater than 25 kDa in size, have modular organization defined by two distinct domains: N-terminal domain and C-terminal binding domain, corresponding to the two functions of enzymatic hydrolysis and substrate recognition. In general, bacteriophage lysins quickly break the cell wall of host bacteria, especially the Gram-positive bacteria. However, the access of lysins to the peptidoglycan in Gram-negative bacteria can be problematic because the outer membrane can block their entry and, as a result, their efficiency is limited [20]. Recently, more research has focused on lysin enzymes such as lysin Cpl-1 to treat pneumococcal infection [22,23], PlySs2 to treat *Streptococcus pyogenes* and *Staphylococcus aureus* [24]; PlyCD for *Clostridium difficile* [25]; PlyE146 for *E*. *coli* K12, *Pseudomonas aeruginosa,* and *Acinetobacter baumannii* [26]; PlyF307 for *Acinetobacter baumannii* [27]; and ABgp46 for *Acinetobacter baumannii, Pseudomonas aeruginosa,* and *Salmonella typhimurium* [28].

Development of bacteriophage biocontrol methods as a means of treating *Vibrio* spp. contamination in aquaculture systems is attractive for three main reasons: (1) Most bacteria are parasitized by at least one bacteriophage and the bacteriophage plays an important role in the evolution of the host genome; (2) land and water resources host large numbers of bacteriophages, estimated at 10–100 times greater than that of the host bacteria; and (3) the potential of bacteriophage therapy as an alternative treatment to antibiotics [29]. However, the effectiveness and potential application of bacteriophage therapy for reduction of *Vibrio* spp. in oyster hatcheries has not yet been extensively studied. The objectives of this study were, therefore, to isolate pathogenic *Vibrio* spp. and their specific bacteriophages, and to determine the effectiveness of a bacteriophage cocktail composed of these isolated bacteriophages against *Vibrio* spp. infesting cultures of oyster larvae. Moreover, characteristics of the isolated bacteriophages, including a full genome analysis, were also investigated.

## 2. Results

### 2.1. Vibrio spp. Isolation and Pathogenicity Test

**Figure 1:**
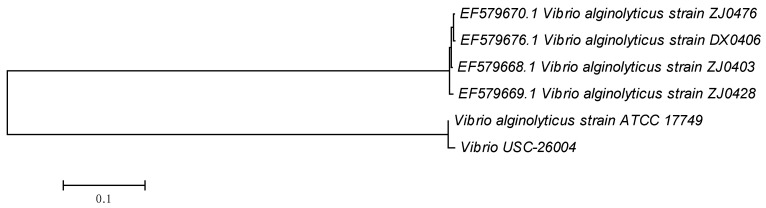
Phylogenetic tree of the pathogenic *Vibrio* sp. (USC-26004) in relation to its closest relatives.

Conventional biochemical tests revealed that the isolates USC-26004 and USC-26005 were *V. alginolyticus* (98.2%) and *V. alginolyticus* (98.6%)*,* respectively. The isolate USC-26004 (Figure 1) caused 100% mortality of oyster larvae within 36 h with a pathogenicity rate of 17.7%. In contrast, the pathogenicity rate of USC-26005 was 2.5% (Table 1).

### 2.2. Prophage Detection in Bacterial Isolates and Antibiotic Susceptibility

The concentration of bacterial isolates increased over the incubation period. No plaque from the spot technique was seen (results not shown). Therefore, there was no prophage detected in the isolated *Vibrio* USC-26005 and USC-26004′s chromosomes.

*Vibrio* isolate USC-26004 was found to be resistant to aztreonam, erythromycin, gentamicin, streptomycin, cefoxitin, and ampicillin and to have intermediate-resistance to imipenem.

### 2.3. Isolation and Characterization of Bacteriophages

**Figure 2:**
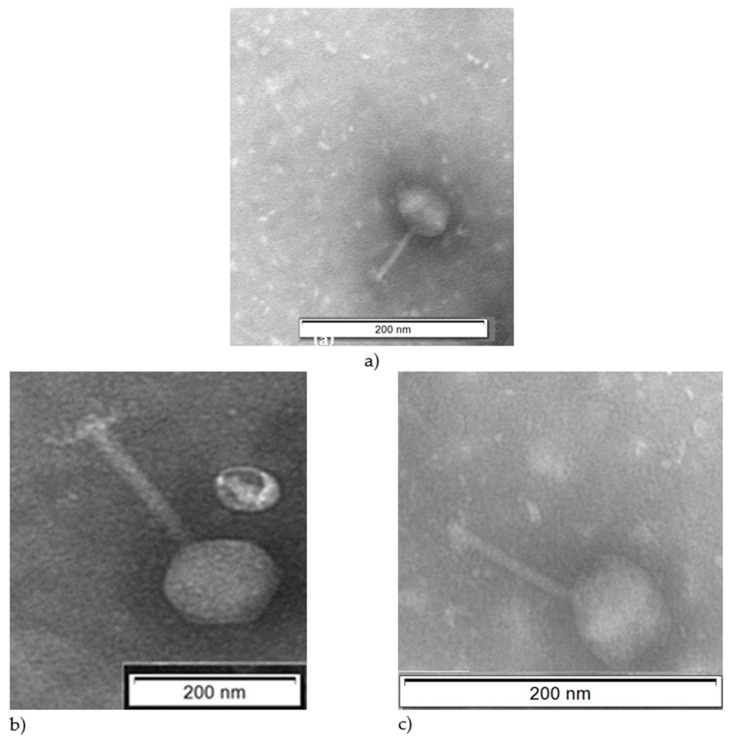
Transmission electron micrographs of the *Vibrio* bacteriophages isolated in this study. (**a**) Φ-5, (**b**) Φ-6, (**c**) Φ-7.

Three different bacteriophages namely Φ-5, Φ-6, and Φ-7 were isolated and examined using transmission electron microscope (TEM) which showed that all bacteriophages had long tails. Characteristics of these bacteriophages are shown in Figure 2 and Table 2.

S1 nuclease and RNase A endonuclease digestion did not result in the production of fragments or degraded genomic DNA for any of the three isolated bacteriophages (results not shown), therefore, it was concluded that the nucleic acids of all bacteriophage isolates were double stranded DNA.

Using combined results from the TEM imaging and the digestions of genomic DNA, the Φ-5, Φ-6, and Φ-7 were deduced to belong to the *Myoviridae* viral family (Table 2).

Genomes of Φ-5 were aligned with toxic *Vibrio* genes: *toxR*, *toxS* gene (GenBank of AB372519.1), *tlh* gene (GenBank number of JQ929914.1), *ctxA* (GenBank number of FJ641050.1 and FJ641047.1), *tcpA* and *tcpB* (GenBank number of AF516340.1) using Blastn suite (Align Sequences Nucleotide BLAST) on https://blast.ncbi.nlm.nih.gov/Blast.cgi. The results revealed that there was no significant similarity between toxic genes located on the genome of Φ-5. The annotation of the genomes of the Φ-5 indicated that no integrase, transposase or repressor genes were present.

Φ-5, which had strong lytic activity, was chosen for genome sequencing and it was found that Φ-5 consisted of 238053 bp with an average GC content of 43.57%. There was no ncRNA gene in the genome and a total of 217 ORFs were predicted for *Vibrio* Φ-5 (Table 2). ORF 46, predicted as lysin, showed high level sequence similarity (95%) to other previously described bacteriophage lysins. Putative thymidine kinase (92% identity), which is used in many antiviral drugs, was found in ORF 10 (Table S1).

Multiple sequence alignment revealed that Φ-5 was closely related to *Vibrio* bacteriophage Aphrodite1 (GenBank number MG720308.1) with 95% similarity and belonged to the genus *Aphroditevirus*.

**Figure 3:**
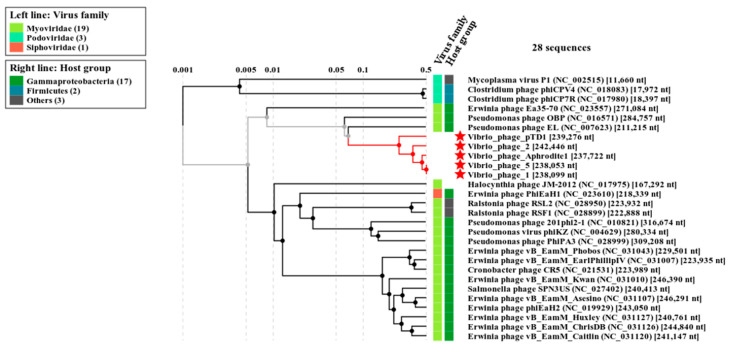
Bacteriophage proteomic tree.

A proteomic tree (Figure 3) was produced based on TBLASTX genomic sequence of 26 bacteriophage genomes. The lengths of branches were logarithmically scaled. The tree was drawn by using the method of Nishimura et al. [31] (https://www.genome.jp/viptree/). *Vibrio* bacteriophage pTD1 and *Vibrio* bacteriophage Aphrodite1 has NCBI accession numbers of AP017972.1 and MG720308.1, respectively. *Vibrio* phage 2 and *Vibrio* phage 1 were isolated and stored at USC lab.

**Figure 4:**
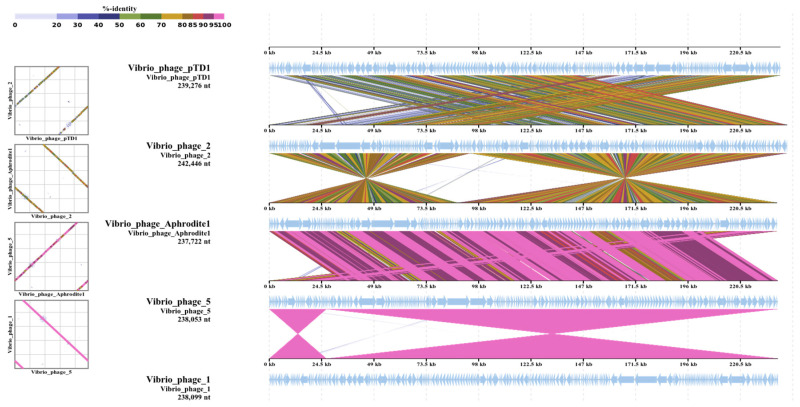
Analysis of the comparative genomes of the *Vibrio* bacteriophages.

Color bar (Figure 4) shows % identity of TBLASTX. The comparative genomes were drawn by using a method of Nishimura et al. [31] (https://www.genome.jp/viptree/). *Vibrio* bacteriophage pTD1 and *Vibrio* bacteriophage Aphrodite1 has GenBank numbers of AP017972.1 and MG720308.1, respectively. *Vibrio*_phage_2 and *Vibrio*_phage_1 were isolated and stored at USC lab.

### 2.4. Host Range and Bacteriophage Lytic Ability

Φ-6 and Φ-7 showed cross infectivity against *V. harveyi* (ATCC 14126) and *V. alginolyticus* (ATCC 17749), respectively (Table 3). Both isolated *Vibrio* spp. were susceptible to the bacteriophages.

**Figure 5:**
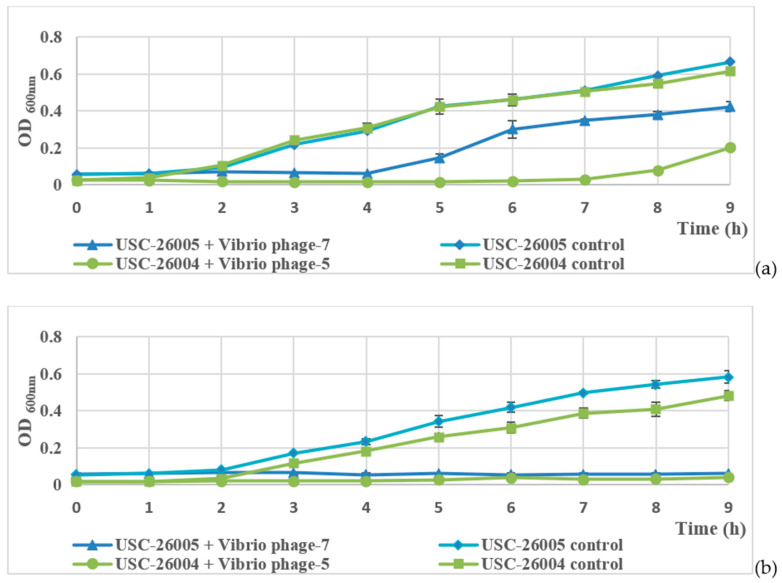
Inactivation of *Vibrio* spp. by the bacteriophages. Initial concentrations of Φ-6 and Φ-7 were ×10^6^ PFU/mL. (**a**) LB broth: The initial concentrations of the isolates USC-26005 + Φ-7, USC-26006 control, USC-2004 + Φ-5 and USC-2004 control were 6.21 ± 0.05 × 10^6^, 6.11 ± 0.09 × 10^6^, 4.04 ± 0.2 × 10^6^ and 4.04 ± 0.2 × 10^6^ CFU/mL, respectively. (**b**) A mixture of seawater and LB broth at a ratio of 3:1: The initial concentrations of the isolates USC-26005 + Φ-7, USC-26006 control, USC-2004 + Φ-5 and USC-2004 control were 6.24 ± 0.1 × 10^6^, 5.92 × 10^6^, 3.62 ± 0.05 × 10^6^ and 3.59 ± 0.12 × 10^6^ CFU/mL, respectively.

In Luria-Bertani (LB) broth, concentrations of USC-26005 and USC-26004 increased gradually and reached average OD_600nm_ values of 0.665 and 0.677, respectively, during the 9 h incubation. Application of the *Vibrio* bacteriophages resulted in a high inhibition of bacterial growth. After 4 h for Φ-7 treatment and 7 h for Φ-5 treatment, bacterial growth recovered and reached much lower OD_600nm_ values compared to the untreated control (Figure 5a).

In seawater, during infection with Φ-5 and Φ-7, the OD value of isolated USC-26005 and USC-26004 remained stable at around 0.06 and 0.02. At the end of this experiment, the OD_600nm_ value of USC-26005 control and USC-26004 control were at the highest levels of 0.584 ± 0.032 and 0.481 ± 0.027, respectively (Figure 5b).

### 2.5. Laboratory Trial of Bacteriophages Against Vibrio spp.

**Figure 6:**
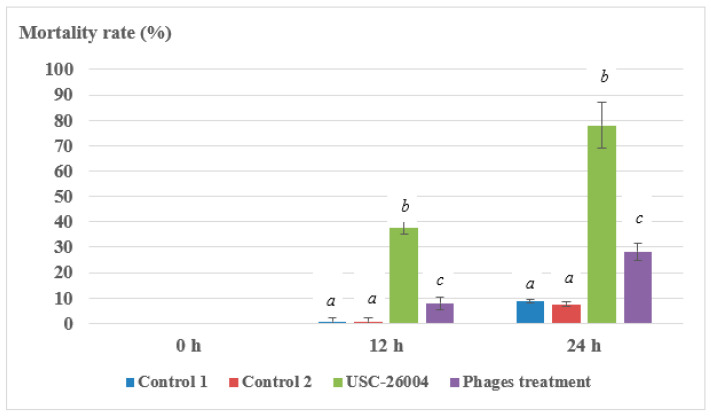
Mortality rate (%) of oyster (*Saccostrea glomerata*) larvae when challenged with USC-26004 and treated with a bacteriophage cocktail. The bacteriophage cocktail was composed of a 1:1:1 mixture of the bacteriophages Φ-5, Φ-6 and Φ-7. Control 1 (mixed phages cocktail), control 2 (larvae only with sterile seawater). Letters (a, b, and c) donate statistically significant differences between treatments versus time among the same types of applications.

There was no significant difference between control 1 and control 2 (*p* > 0.05), indicating that the bacteriophage cocktail did not affect oyster larval health. Mortality rate (%) of larvae was 28.2 ± 3.5 in the bacteriophage treatment group, compared to 77.9 ± 9.1 in the bacterial treatment group after 24 h incubation (Figure 6).

## 3. Discussion

Several *Vibrio* spp., including *V. alginolyticus*, are considered to be the most significant aquaculture pathogens causing mortality of shellfish and crustaceans. Three antibiotics (oxytetracycline, chloramphenicol and oxolinic acid) are commonly used in aquaculture [2]. Watts et al. [32] pointed out that 90% of bacteria isolated from seawater were resistant to at least one antibiotic and 20% of bacteria were resistant to five or more antibiotics. Kang et al. [14] reported on the pathogenic *V. alginolyticus* that caused severe infection in shellfish in coastal areas of Korea where all of 15 isolates tested were resistant to vancomycin and ampicillin and 33% of *Vibrio* isolates showed multiple antimicrobial resistance to at least three antibiotics. Even though the use of antibiotics in some developed countries such as Japan, North America, and Europe is strictly regulated or banned, there is a lack of regulation on antibiotics use in developing nations where 90% of world aquaculture production occurs. This results in high variability in the use of antibiotics in different regions and the potential accumulation of antibiotic residues in the produce. Antibiotic residues in farmed salmon for example, ranged from 0.02–0.39 g/ton in Scotland and Norway, to 660 g/ton in Chile [32]. In line with the above information, the most pathogenic isolate in the present study was USC-26004 which also displayed antibiotic resistant properties. Antibiotic resistance was also observed for *Vibrio* isolates similar to those observed by Kang et al. [14] and Karunasagar et al. [12], indicating that antibiotic resistant *Vibrio* species may have increasing prevalence in the environment. Antibiotic resistance also occurs in aquaculture systems that are considered “hotspots for antibiotic resistance genes” where recombination and gene exchange can easily occur [32]. As a result, bacteriophage control that also eliminates the possibility of antibiotic resistant bacteria, presents an effective potential control strategy.

Ackermann [33] reported that from a total of 227 tailed *Vibrio* bacteriophages, 67 (29.5%) and 86 (37.9%) belonged to the *Siphoviridae* and *Myoviridae* viral families, respectively. In the present study, the *Vibrio* bacteriophage isolates were also classified into the *Myoviridae* viral family. Moreover, the sizes of the *Vibrio* bacteriophages corresponded to those reported by Ackermann [34], where capsid and tail size of tailed bacteriophages were from 30 to 160 and 10 to 800 nm, respectively. The three bacteriophage genomes could not be digested by S1 nuclease or RNase A, confirming that they were all double stranded DNA bacteriophages, and again, these findings were consistent with results from Sasikala and Srinivasan [35]. The genome of Φ-5 was relatively large, compared to PVA1 *Vibrio alginolyticus* bacteriophage, in which the genomic sequence contained 41529 bp with a GC content of 43.7% [36].

For acceptance of the use of bacteriophages as biological control agents in aquaculture, better understanding of the genetics and the biology of bacteriophages is required. One of the potential obstacles can be related to temperate bacteriophages, that can transduce virulence factors to host bacteria [37]. Many researchers have reported that bacteriophages can encode toxins or other toxicity factors which could increase the virulence of their host bacteria. This obstacle could be removed by selecting virulent bacteriophages. Moreover, in order to be an effective biological control candidate, the genome of selected bacteriophages should not contain any of the known virulence-associated or antibiotic-resistance genes [37]. Therefore, bacteriophage genome analysis has to be carried out to determine that none of the putative proteins would have significant similarities to hypothetical factors or genes known to play roles in bacterial pathogenicity. The annotation of the genome of Φ-5 indicated that no integrase, transposase or repressor genes were present in the genome. Therefore, the truly lytic nature of Φ-5 implied that it is a suitable candidate for biological control of *V. alginolyticus* (USC 26001, USC 26004, ATCC 17749). Another potential obstacle to the use of bacteriophages as biocontrol agents is to ensure that they do not perform generalized or specialized transduction [38]. Based on the genome analysis and the subsequent growth experiments, it was concluded that the bacteriophages described in this paper were unlikely to be involved in the specialized transduction of host bacterial DNA. The GC content of Φ-1 was very close to that for bacteriophage *V. alginolyticus* Vp 670 (GC content of 43.4%) [39] with no ncRNA gene in the genome [38]. Bacteriophages with tails and a dsDNA genome larger than 200 kb are classified as Jumbo bacteriophage [40]. Φ-5 is a jumbo bacteriophage. Similar to other Jumbo phages, which are often isolated from aquatic environments, they mostly infect Gram-negative host bacteria (95.6%) [40].

Until now, 578 *Vibrio* bacteriophage genomes have been sequenced (http://millardlab.org/bioinformatics/bacteriophage-genomes/phage-genomes-april-2019/) and the data obtained have paved the way to new approaches relating to the utilization of *Vibrio* bacteriophage lysins as potential antimicrobial agents. However, due to the outer-membrane protection of Gram-negative bacteria, lysins can only act effectively against Gram-positive bacteria and they work less effectively against Gram-negative bacteria. Lysins might be more effective against Gram-negative bacteria if the bacteria were pretreated with the outer membrane proteins (CHCl_3_, EDTA or Triton X-100) [41,42,43,44]. In order to apply in aquaculture settings, large volume and cost-effective scale-up of lysin production is required. These factors are currently significant obstacles against cost-effective implementation of the use of lysins for disease control in aquaculture. Therefore, in this study, phage lysin was not investigated.

Further studies could be conducted to determine whether or not bacteriophage treatments would be effective in aquaculture facilities. Moreover, the performance of bacteriophages under the range of environmental conditions that might be experienced in aquaculture systems needs to be investigated to enable generation of an improved understanding of appropriate and sub-optimal conditions under which bacteriophage treatment might be successfully applied. Further research should also be carried out on bacteriophage-resistant bacteria to confirm that mutant bacteria are not likely to be pathogenic [45].

## 4. Materials and Methods

### 4.1. Isolation and Characterization of Vibrio spp. Isolated from Oyster Hatcheries

*Vibrio* spp. were isolated from water within larval culture tanks at an oyster hatchery (Port Stephens Fisheries Institute, New South Wales, Australia), by concentrating samples from the cultures on Whatman filter papers (Merck, Macquarie Park NSW, Australia) followed by an enrichment. Isolates were then identified using conventional biochemical tests in the form of commercial kits API^®^20NE (BioMerieux, Durham, NC 27712, USA) (https://www.biomerieux-usa.com/clinical/api). After incubation, colony morphology on the agar plates was examined, isolations were conducted, and the resulting pure bacterial cultures were stored at −80 °C in the DMB supplemented with 15% glycerol.

The DNA of isolated bacteria was extracted according to the manufacturer’s instructions, using the GenElute bacterial genomic DNA kit (Merck, Macquarie Park NSW, Australia). Universal primers U1492R and Bac27F [46] were used to amplify the partial 16S rDNA gene, using thermal cycles, based on the method of Pang et al. [47]. The resulting PCR products were sequenced by the Macrogen Company in the Republic of Korea (http://www.macrogen.com/en/main/index.php) and the sequences were subsequently analyzed using BLAST analysis (https://blast.ncbi.nlm.nih.gov). The resulting sequence of USC-26004 was deposited in Genbank (accession number of MK334309).

The disk diffusion method [48] on Müller–Hinton agar (Thermo Scientific, Scoresby, Vic, Australia) was used to determine the antibiotic resistance patterns of the *Vibrio* isolates against the following antimicrobials: aztreonam (30 µg), tetracycline (30 µg), nalidixic acid (30 µg), cefoxitin (30 µg), erythromycin (15 µg), gentamicin (10 µg), streptomycin (10 µg), ampicillin (10 µg), imipenem (10 µg), and penicillin (10 units) (Edwards Group Pty. Ltd., NSW, Australia). Plates were incubated for 24 h at 37 °C. Because no breakpoints are defined for *Vibrio*, the zone diameter interpretive standards for the *Enterobacteriaceae* [48] was used in this study and the *E. coli* strain (ATCC 25922) was used as the control during the test.

### 4.2. Pathogenicity Test and Prophage Induction

All isolated *Vibrio* spp., *V. harveyi* (ATCC 14126), and *V. alginolyticus* (ATCC 17749) were tested for their possible pathogenicity against oyster larvae using the method described by Prado et al. [30]. Healthy Sydney rock oyster (*Saccostrea glomerata*) larvae (200 µm size antero-posterior shell size) were used at a density of 8–12 larvae/mL in 2.5 mL wells (total of 24–30 larvae per well) and the final concentration of *Vibrio* spp. administered to larval cultures was ~10^5^ CFU/mL. Mortalities lower than 10% indicated that the bacteria were not pathogenic [30]. All tested groups were performed in triplicate.

The prophage induction test of *Vibrio* spp. was carried out as described by Clokie and Kropinski [49].

### 4.3. Isolation and Characterization of Bacteriophages

Bacteriophage isolation and genome sequencing was carried out as described by Clokie and Kropinski [49].

Rectangular proteomic tree and genomic alignment were created by using method of Nishimura et al. [31].

Genome of Φ-5 was aligned with toxic *Vibrio* genes: toxR, toxS gene (GenBank of AB372519.1), tlh gene (GenBank number of JQ929914.1), ctxA (GenBank number of FJ641050.1 and FJ641047.1), tcpA and tcpB (GenBank number of AF516340.1) using Blastn suite (Align Sequences Nucleotide BLAST) on https://blast.ncbi.nlm.nih.gov/Blast.cgi.

The GenBank numbers of Φ-1 (or *Vibrio* phage USC-1) and Φ-5 were MK905543 and MK358448, respectively.

### 4.4. Bacteriophage Lytic Activity

*Vibrio* species were streaked onto Tryptone Soya Agar with 1.5% salt agar (TSAS) and incubated at 28 °C overnight, then harvested onto LB broth (Thermo Fisher Scientific, Scoresby, VIC, Australia). An aliquot (6 mL) of the *Vibrio* spp. in LB broth (~10^6^ CFU/mL) was added into 2 mL of a bacteriophage solution (concentration of ~10^6^ to ~10^8^ PFU/mL) and incubated at 28 °C and 150 rpm. A control using only *Vibrio* spp. was also included in the test. Samples were removed from the broth every hour for 9 h and the optical density of the cells measured at 600 nm. A similar experiment to that as detailed above was carried out with the exception that the LB broth was replaced with sterile seawater to measure bacteriophage lytic activity in a mixture of sterile seawater and LB broth at a ratio of 3:1. All tests were performed in triplicate.

### 4.5. Bacteriophage Control of Vibrio spp. with Oyster Larvae

*Vibrio* sp. (USC-26004) was used for challenge tests in 12-well sterile tissue cell-culture plates (Thermo Fisher Scientific, Scoresby, VIC, Australia) [30,50]. Oyster *(S. glomerata)* larvae with an antero–posterior shell size of around 200 µm, were placed at a stocking density of 8–12 larvae/mL into each 2.5 mL well (total of 24–30 larvae per well). A bacteriophage cocktail composed of a 1:1:1 mixture of the bacteriophages Φ-5, Φ-6 and Φ-7 was prepared. The challenge included a positive control group (larvae with exposure to USC-26004), a bacteriophage control 1 (mixed phages cocktail), control 2 (larvae only with sterile seawater) and a bacteriophage treated group. An aliquot (0.1 mL) of mixed bacteriophage cocktail (final concentration of 10^8^ PFU/mL) was first added at 0 h and then added with another 0.1 mL of the same bacteriophage suspension after 12 h. *Vibrio* USC-26004 was added to give a final concentration of 10^5^ CFU/mL in the well. The challenges were run for a period of 24 h. Larval mortality was determined every 12 h using a light microscope (4×–10×) and mortality was characterized by the lack of identifiable movement of larval tissues. Larvae were fed every 12 h during the challenge test with live microalgae (*Tetraselmis chuii*, *Dunaliella tertiolecta* and *Nannochloropsis oculata*) following standard culture procedure for Sydney rock oyster larvae(https://www.dpi.nsw.gov.au/__data/assets/pdf_file/0014/261041/Output-1084_Hatchery-Manual_FORMATTED.pdf). All tested groups were performed in triplicate.

### 4.6. Statistical Analysis

Single factor ANOVA was used to test for differences in larval mortality rates in treatments with and without the bacteriophage suspension. Data were analyzed using SPSS Statistics 20 software (IBM SPSS Statistics 20, USA) (https://www-01.ibm.com/support/docview.wss?uid=swg24029274) and difference was considered significant at *p* < 0.05.

## 5. Conclusions

A bacteriophage cocktail composed of three newly isolated bacteriophages investigated in this study successfully reduced levels of *Vibrio* spp. contamination and improved the survival of oyster larvae exposed to pathogenic *Vibrio* spp. On this basis, bacteriophage treatment may be regarded as having potential as an effective biocontrol agent that prevents the onset of vibriosis in oyster hatcheries and, potentially, in other aquaculture settings. The genome of Φ-5 was found to contain lysin genes. Findings presented increase our understanding of bacteriophage biology, genomics, and bacteriophage lysins, and they highlight the potential of bacteriophage biocontrol as an alternative treatment for vibriosis in aquaculture facilities.

## Figures and Tables

**Table 1:** Recorded mortality (%) of oyster (*Saccostrea glomerata*) larvae in challenge tests when exposed to different *Vibrio* isolates.

*Vibrio* Species	BLAST (Identity %)	Pathogenicity Rate (%)
USC-26004	*V. alginolyticus* (99%)	17.7
USC-26005	*V. alginolyticus* (99%)	2.5
ATCC 14126	*V. harveyi*	16.6
ATCC 17749	*V. alginolyticus*	12.9

Data presented as averages. All tested groups were performed in triplicate using the method of Prado et al. [30].

**Table 2:** Characteristics of the *Vibrio* bacteriophages isolated in this study.

Bacteriophage	Head (nm)		Nucleic Acid	Neck (nm)		Tail Sheath (nm)		Family
	L	W		L	W	L	W	
Φ-5	131.8	122.7	DNA, ds	11.4	6.8	129.5	18.2	*Myoviridae*
Φ-6	NM	96.0	DNA, ds	NM	NM	162.2	16.1	*Myoviridae*
Φ-7	68.6	59.1	DNA, ds	4.76	3.8	81.9	8.6	*Myoviridae*

The concentration of bacteriophages was ~×10^8^ PFU/mL; W: wide; L: length, NM: not measured. Data presented as averages. Bacteriophages were measured in triplicate.

**Table 3:** Host range of the bacteriophage isolates determined against different *Vibrio* species.

*Vibrio* spp.	*Vibrio* Φ-5	*Vibrio* Φ-6	*Vibrio* Φ-7
USC-26004	+	+	+
USC-26005	PH	PH	PH
ATCC 14126	-	+	-
ATCC 17749	-	-	+

“+”: susceptible to bacteriophage lysis; “-”: not susceptible to bacteriophage lysis; PH: propagation host.

## Supplementary Materials

The following are available online at https://www.mdpi.com/2079-6382/9/7/415/s1, Table S1: Predicted ORFs for Φ-5 and homology to proteins.
