# Supplementary material for: Application of Bacteriophages to Control Vibrio alginolyticus Contamination in Oyster (Saccostrea glomerata) Larvae
Source: Antibiotics (Basel). 2020 Jul 16;9(7):415. doi: 10.3390/antibiotics9070415 (PMC7400271; doi:10.3390/antibiotics9070415)
Supplement: Supplementary file 1 [file antibiotics-09-00415-s001.pdf]

Supplementary material

Application of bacteriophages to control *Vibrio alginolyticus* contamination in oyster (*Saccostrea glomerata*) larvae

Tuan Son Le[1,4], Paul C. Southgate[2], Wayne O'Connor[3], Sang V. Vu [4], D. İpek Kurtböke[4,*]

1 Research Institute for Marine Fisheries, 224 Le Lai, Ngo Quyen, Hai Phong 180000, Vietnam; (letuanson1987@gmail.com; Tuan.Son.Le@research.usc.edu.au)
2 Australian Centre for Pacific Islands Research and School of Science and Engineering, University of the Sunshine Coast, Maroochydore, Queensland 4556, Australia. Paul C. Southgate (psouthgate@usc.edu.au) and Sang V. Vu (vuvansangts50@gmail.com; v_v013@student.usc.edu.au)
3 NSW Fisheries, Port Stephens Fisheries Institute, Taylors Beach, NSW 2316, Australia, (wayne.oconnor@dpi.nsw.gov.au)
4 GeneCology Research Centre and School of Science and Engineering, University of the Sunshine Coast, 90 Sippy Downs Drive, Sippy Downs, Queensland 4556, Australia.
* Correspondence: ikurtbok@usc.edu.au ; Tel.: +61-7-5430-2918

Table S1. Predicted ORFs for *Vibrio* phage 5 and homology to proteins

ORFs	Position		Size		Stand	Predicted function	E value	Identity (%)	Accession
	Start	End	bp	aa					
1	87	1055	969	322	+	hypothetical protein Aphrodite1_0039 [*Vibrio* phage Aphrodite1]	1.00E-151	64%	AUR80987.1
2	1080	1604	525	174	+	ribonuclease HI [*Vibrio* phage Aphrodite1]	1.00E-124	98%	AUR81037.1
3	1601	1921	321	106	+	hypothetical protein Aphrodite1_0037 [*Vibrio* phage Aphrodite1]	2.00E-66	88%	AUR81089.1
4	1986	2477	492	163	+	hypothetical protein Aphrodite1_0036 [*Vibrio* phage Aphrodite1]	7.00E-114	97%	AUR81047.1
5	2488	3132	645	214	+	hypothetical protein Aphrodite1_0035 [*Vibrio* phage Aphrodite1]	8.00E-151	94%	AUR81022.1
6	3135	4280	1146	381	+	hypothetical protein Aphrodite1_0034 [*Vibrio* phage Aphrodite1]	0	97%	AUR80973.1

7	4353	5912	1560	519	+	heat shock protein 60 family chaperone [*Vibrio* phage Aphrodite1]	0	99%	AUR80941.1
8	6020	6478	459	152	+	hypothetical protein CP-T1_0029 [*Vibrio* phage CP-T1]	0.013	37%	YP_007003069.1
9	6903	7499	597	198	+	thymidine kinase [*Vibrio* phage Aphrodite1]	7.00E-136	92%	AUR81030.1
10	7557	8744	1188	395	+	hypothetical protein [*Vibrio* phage pTD1]	2.00E-120	47%	BAW98230.1
11	8815	12498	3684	1227	+	hypothetical protein Aphrodite1_0027 [*Vibrio* phage Aphrodite1]	0	60%	AUR80912.1
12	12609	13379	771	256	+	hypothetical protein Aphrodite1_0026 [*Vibrio* phage Aphrodite1]	2.00E-174	93%	AUR81007.1
13	13427	14446	1020	339	+	hypothetical protein Aphrodite1_0025 [*Vibrio* phage Aphrodite1]	0	93%	AUR80983.1
14	14450	14782	333	110	+	hypothetical protein Aphrodite1_0024 [*Vibrio* phage Aphrodite1]	7.00E-72	95%	AUR81083.1
15	14875	15327	453	150	+	hypothetical protein Aphrodite1_0023 [*Vibrio* phage Aphrodite1]	4.00E-104	96%	AUR81056.1
16	15375	15872	498	165	+	hypothetical protein Aphrodite1_0022 [*Vibrio* phage Aphrodite1]	3.00E-115	95%	AUR81044.1
17	15874	16344	471	156	+	hypothetical protein Aphrodite1_0021 [*Vibrio* phage Aphrodite1]	6.00E-110	97%	AUR81053.1
18	16364	16855	492	163	+	hypothetical protein Aphrodite1_0020 [*Vibrio* phage Aphrodite1]	3.00E-114	100%	AUR81046.1
19	16919	18991	2073	690	-	DNA helicase [*Vibrio* phage Aphrodite1]	0	99%	AUR80929.1
20	19049	20404	1356	451	+	hypothetical protein Aphrodite1_0018 [*Vibrio* phage Aphrodite1]	0	99%	AUR80955.1
21	20457	20747	291	96	+	hypothetical protein Aphrodite1_0017 [*Vibrio* phage Aphrodite1]	7.00E-64	99%	AUR81095.1
22	20878	21633	756	251	+	none			
23	21643	23166	1524	507	+	hypothetical protein Aphrodite1_0015 [*Vibrio* phage Aphrodite1]	0	98%	AUR80944.1
24	23214	24302	1089	362	+	hypothetical protein Aphrodite1_0014 [*Vibrio* phage Aphrodite1]	0	98%	AUR80978.1
25	24292	24588	297	98	+	hypothetical protein Aphrodite1_0013 [*Vibrio* phage	9.00E-67	100%	AUR81094.1

						Aphrodite1]			
26	24645	25157	513	170	+	hypothetical protein Aphrodite1_0012 [*Vibrio* phage Aphrodite1]	5.00E-122	99%	AUR81039.1
27	25164	25943	780	259	+	hypothetical protein Aphrodite1_0011 [*Vibrio* phage Aphrodite1]	0	100%	AUR81004.1
28	25940	26617	678	225	+	hypothetical protein Aphrodite1_0010 [*Vibrio* phage Aphrodite1]	8.00E-146	89%	AUR81018.1
29	26628	27239	612	203	+	hypothetical protein Aphrodite1_0009 [*Vibrio* phage Aphrodite1]	3.00E-89	62%	AUR81028.1
30	27229	27558	330	109	+	hypothetical protein Aphrodite1_0008 [*Vibrio* phage Aphrodite1]	6.00E-72	94%	AUR81084.1
31	27619	28254	636	211	-	hypothetical protein Aphrodite1_0007 [*Vibrio* phage Aphrodite1]	3.00E-151	99%	AUR81023.1
32	28247	29575	1329	442	-	putative virion structural protein [*Vibrio* phage Aphrodite1]	0	100%	AUR80957.1
33	29616	30047	432	143	+	hypothetical protein Aphrodite1_0005 [*Vibrio* phage Aphrodite1]	9.00E-99	99%	AUR81063.1
34	30140	31672	1533	510	+	hypothetical protein Aphrodite1_0004 [*Vibrio* phage Aphrodite1]	0	99%	AUR80943.1
35	31711	32553	843	280	-	hypothetical protein Aphrodite1_0003 [*Vibrio* phage Aphrodite1]	0	97%	AUR81001.1
36	32577	34013	1437	478	+	hypothetical protein Aphrodite1_0002 [*Vibrio* phage Aphrodite1]	0	99%	AUR80950.1
37	34074	34439	366	121	+	hypothetical protein Aphrodite1_0001 [*Vibrio* phage Aphrodite1]	1.00E-81	98%	AUR81077.1
38	34441	35121	681	226	+	putative virion structural protein [*Vibrio* phage Aphrodite1]	2.00E-149	99%	AUR81025.1
39	35123	35635	513	170	+	hypothetical protein Aphrodite1_0208 [*Vibrio* phage Aphrodite1]	1.00E-107	88%	AUR81040.1
40	35637	36371	735	244	+	hypothetical protein Aphrodite1_0207 [*Vibrio* phage Aphrodite1]	1.00E-170	96%	AUR81010.1
41	36431	36934	504	167	-	hypothetical protein Aphrodite1_0206 [*Vibrio* phage Aphrodite1]	1.00E-117	99%	AUR81043.1
42	36998	37612	615	204	+	hypothetical protein Aphrodite1_0205 [*Vibrio* phage	5.00E-143	98%	AUR81029.1

						Aphrodite1]			
43	37581	37994	414	137	+	hypothetical protein Aphrodite1_0204 [*Vibrio* phage Aphrodite1]	3.00E-88	99%	AUR81070.1
44	38090	39220	1131	376	+	hypothetical protein Aphrodite1_0203 [*Vibrio* phage Aphrodite1]	0	97%	AUR80974.1
45	39273	41447	2175	724	-	hypothetical protein Aphrodite1_0202 [*Vibrio* phage Aphrodite1]	0	97%	AUR80924.1
46	41499	48479	6981	2326	-	endolysin [*Vibrio* phage Aphrodite1]	0	95%	AUR80910.1
47	48502	50109	1608	535	+	hypothetical protein Aphrodite1_0200 [*Vibrio* phage Aphrodite1]	0	99%	AUR80938.1
48	50112	54461	4350	1449	+	putative RNA polymerase beta subunit [*Vibrio* phage Aphrodite1]	0	98%	AUR80913.1
49	54465	55319	855	284	+	hypothetical protein Aphrodite1_0198 [*Vibrio* phage Aphrodite1]	0	96%	AUR80998.1
50	55319	55480	162	53	+	hypothetical protein Aphrodite1_0197 [*Vibrio* phage Aphrodite1]	5.00E-27	100%	AUR81099.1
51	55483	56973	1491	496	+	hypothetical protein Aphrodite1_0196 [*Vibrio* phage Aphrodite1]	0	97%	AUR80945.1
52	56990	57724	735	244	+	hypothetical protein Aphrodite1_0195 [*Vibrio* phage Aphrodite1]	4.00E-175	98%	AUR81009.1
53	57717	58610	894	297	+	putative virion structural protein [*Vibrio* phage Aphrodite1]	0	99%	AUR80994.1
54	58719	59384	666	221	+	hypothetical protein Aphrodite1_0193 [*Vibrio* phage Aphrodite1]	5.00E-158	98%	AUR81019.1
55	59497	59943	447	148	+	hypothetical protein Aphrodite1_0192 [*Vibrio* phage Aphrodite1]	1.00E-104	99%	AUR81057.1
56	60091	60534	444	147	+	hypothetical protein Aphrodite1_0191 [*Vibrio* phage Aphrodite1]	6.00E-92	90%	AUR81060.1
57	60604	60717	114	37	+	hypothetical protein Aphrodite1_0190 [*Vibrio* phage Aphrodite1]	1.00E-16	100%	AUR81105.1
58	60714	61196	483	160	+	hypothetical protein Aphrodite1_0189 [*Vibrio* phage Aphrodite1]	5.00E-112	99%	AUR81050.1
59	61199	61858	660	219	+	hypothetical protein Aphrodite1_0188 [*Vibrio* phage	6.00E-157	96%	AUR81020.1

						Aphrodite1]			
60	61855	62337	483	160	+	hypothetical protein Aphrodite1_0187 [*Vibrio* phage Aphrodite1]	3.00E-112	99%	AUR81049.1
61	62334	62852	519	172	+	hypothetical protein Aphrodite1_0186 [*Vibrio* phage Aphrodite1]	3.00E-118	97%	AUR81038.1
62	62921	63370	450	149	+	hypothetical protein Aphrodite1_0185 [*Vibrio* phage Aphrodite1]	2.00E-108	100%	AUR81051.1
63	63505	64293	789	262	+	putative metal-dependent phosphohydrolase [*Vibrio* phage Aphrodite1]	0	99%	AUR81003.1
64	64295	65203	909	302	+	thymidylate synthase [*Vibrio* phage Aphrodite1]	0	96%	AUR80992.1
65	65277	65732	456	151	+	hypothetical protein [*Vibrio* phage VP4B]	3.00E-11	29%	AGB07148.1
66	65733	66371	639	212	+	hypothetical protein [*Vibrio* phage pTD1]	6.00E-18	27%	BAW98383.1
67	66371	67078	708	235	+	hypothetical protein Aphrodite1_0179 [*Vibrio* phage Aphrodite1]	5.00E-42	37%	AUR81015.1
68	67082	67726	645	214	+	hypothetical protein [*Vibrio* phage VP4B]	2.00E-15	25%	AGB07149.1
69	67723	68439	717	238	+	hypothetical protein Aphrodite1_0179 [*Vibrio* phage Aphrodite1]	2.00E-141	86%	AUR81015.1
70	68442	69011	570	189	+	hypothetical protein Aphrodite1_0178 [*Vibrio* phage Aphrodite1]	6.00E-119	86%	AUR81032.1
71	69014	69406	393	130	+	hypothetical protein Aphrodite1_0177 [*Vibrio* phage Aphrodite1]	7.00E-76	89%	AUR81075.1
72	69399	70544	1146	381	+	hypothetical protein [*Vibrio* phage pTD1]	0	82%	BAW98379.1
73	70735	71148	414	137	+	hypothetical protein Aphrodite1_0175 [*Vibrio* phage Aphrodite1]	3.00E-63	79%	AUR81076.1
74	71148	71447	300	99	+	hypothetical protein Aphrodite1_0174 [*Vibrio* phage Aphrodite1]	2.00E-57	86%	AUR81093.1
75	71529	71798	270	89	+	hypothetical protein Aphrodite1_0173 [*Vibrio* phage Aphrodite1]	4.00E-27	63%	AUR81097.1
76	71917	72816	900	299	-	putative tail tube protein [*Vibrio* phage Aphrodite1]	0	100%	AUR80993.1
77	72821	74956	2136	711	-	hypothetical protein Aphrodite1_0171 [*Vibrio* phage Aphrodite1]	0	99%	AUR80926.1

78	75102	75980	879	292	+	putative virion structural protein [*Vibrio* phage Aphrodite1]	0	99%	AUR80997.1
79	76005	78587	2583	860	+	hypothetical protein Aphrodite1_0169 [*Vibrio* phage Aphrodite1]	0	99%	AUR80918.1
80	78580	83343	4764	1587	+	putative virion structural protein [*Vibrio* phage Aphrodite1]	0	89%	AUR80911.1
81	83354	93943	####	3529	+	putative invasin [*Vibrio* phage Aphrodite1]	0	96%	AUR80908.1
82	94091	101743	7653	2550	+	putative invasin [*Vibrio* phage Aphrodite1]	0	92%	AUR80909.1
83	101832	105047	3216	1071	+	hypothetical protein Aphrodite1_0165 [*Vibrio* phage Aphrodite1]	0	97%	AUR80915.1
84	105236	105859	624	207	-	thymidylate kinase [*Vibrio* phage Aphrodite1]	1.00E-151	99%	AUR81027.1
85	105918	107288	1371	456	-	putative virion structural protein [*Vibrio* phage Aphrodite1]	0	99%	AUR80954.1
86	107288	108133	846	281	-	putative virion structural protein [*Vibrio* phage Aphrodite1]	0	99%	AUR80999.1
87	108468	109016	549	182	+	hypothetical protein Aphrodite1_0161 [*Vibrio* phage Aphrodite1]	8.00E-125	95%	AUR81031.1
88	109129	109677	549	182	+	none			
89	109707	110270	564	187	+	secreted hypothetical protein [Pristhesancus plagipennis]	9.4	33%	ATU82816.1
90	110401	110553	153	50	+	none			
91	110943	111335	393	130	+	hypothetical protein Aphrodite1_0158 [*Vibrio* phage Aphrodite1]	4.00E-77	90%	AUR81073.1
92	111353	111823	471	156	+	spore photoproduct lyase [Paenibacillus ginsengarvi]	1	25%	WP_120747463.1
93	111827	112831	1005	334	+	hypothetical protein Aphrodite1_0156 [*Vibrio* phage Aphrodite1]	0	93%	AUR80986.1
94	112982	113326	345	114	+	none			
95	113323	114276	954	317	+	SusC/RagA family TonB-linked outer membrane protein [bacterium A37T11]	3	29%	WP_092390778.1
96	114374	115132	759	252	+	hypothetical protein Aphrodite1_0152 [*Vibrio* phage Aphrodite1]	3.00E-159	83%	AUR81006.1
97	115461	116240	780	259	+	hypothetical protein Aphrodite1_0151 [*Vibrio* phage Aphrodite1]	0	98%	AUR81005.1
98	116312	117490	1179	392	+	hypothetical protein Aphrodite1_0150 [*Vibrio* phage Aphrodite1]	0	96%	AUR80971.1
99	117478	118203	726	241	+	lactose operon transcriptional activator [*Vibrio* phage	7.00E-172	96%	AUR81012.1

						Aphrodite1]			
100	118196	118897	702	233	+	Msm operon regulatory protein [*Vibrio* phage Aphrodite1]	4.00E-131	87%	AUR81017.1
101	119214	119327	114	37	-	none			
102	119396	120361	966	321	+	chain A monomeric subunit of Tubz [*Vibrio* phage Aphrodite1]	0	100%	AUR80988.1
103	120454	120747	294	97	+	hypothetical protein [*Vibrio* phage pTD1]	4.00E-13	37%	BAW98348.1
104	120757	121212	456	151	+	hypothetical protein Aphrodite1_0146 [*Vibrio* phage Aphrodite1]	8.00E-100	94%	AUR81055.1
105	121187	121759	573	190	+	hypothetical protein [*Vibrio* phage VP4B]	7.00E-28	39%	AGB07183.1
106	121746	122057	312	103	+	hypothetical protein Aphrodite1_0145 [*Vibrio* phage Aphrodite1]	8.00E-69	98%	AUR81086.1
107	122047	122373	327	108	+	hypothetical protein Aphrodite1_0144 [*Vibrio* phage Aphrodite1]	5.00E-71	95%	AUR81088.1
108	122351	122680	330	109	+	hypothetical protein Aphrodite1_0143 [*Vibrio* phage Aphrodite1]	2.00E-69	94%	AUR81085.1
109	122710	123117	408	135	+	hypothetical protein Aphrodite1_0142 [*Vibrio* phage Aphrodite1]	7.00E-93	99%	AUR81068.1
110	123184	123621	438	145	+	hypothetical protein Aphrodite1_0141 [*Vibrio* phage Aphrodite1]	1.00E-98	97%	AUR81062.1
111	123680	125752	2073	690	+	hypothetical protein Aphrodite1_0140 [*Vibrio* phage Aphrodite1]	0	99%	AUR80930.1
112	125788	126894	1107	368	-	hypothetical protein Aphrodite1_0139 [*Vibrio* phage Aphrodite1]	0	99%	AUR80977.1
113	127117	129183	2067	688	+	hypothetical protein Aphrodite1_0138 [*Vibrio* phage Aphrodite1]	0	99%	AUR80931.1
114	129278	130765	1488	495	+	putative DNA helicase [*Vibrio* phage Aphrodite1]	0	99%	AUR80946.1
115	131082	132053	972	323	+	hypothetical protein ACD_33C00045G0033 [uncultured bacterium]	1.00E-40	31%	EKD89566.1
116	132099	132287	189	62	+	hypothetical protein Aphrodite1_0136 [*Vibrio* phage Aphrodite1]	1.00E-38	100%	AUR81098.1
117	132287	132943	657	218	+	hypothetical protein Aphrodite1_0135 [*Vibrio* phage Aphrodite1]	2.00E-162	99%	AUR81021.1

118	132969	133445	477	158	+	hypothetical protein Aphrodite1_0134 [*Vibrio* phage Aphrodite1]	1.00E-107	97%	AUR81052.1
119	133519	133647	129	42	+	hypothetical protein Aphrodite1_0133 [*Vibrio* phage Aphrodite1]	1.00E-22	100%	AUR81104.1
120	133711	134133	423	140	+	putative virion structural protein [*Vibrio* phage Aphrodite1]	2.00E-99	100%	AUR81064.1
121	134114	135001	888	295	+	hypothetical protein Aphrodite1_0131 [*Vibrio* phage Aphrodite1]	0	99%	AUR80995.1
122	135023	136147	1125	374	+	hypothetical protein Aphrodite1_0130 [*Vibrio* phage Aphrodite1]	0	99%	AUR80975.1
123	136171	136941	771	256	+	hypothetical protein Aphrodite1_0129 [*Vibrio* phage Aphrodite1]	0	98%	AUR81002.1
124	137015	137731	717	238	+	hypothetical protein Aphrodite1_0128 [*Vibrio* phage Aphrodite1]	5.00E-171	99%	AUR81014.1
125	137845	139419	1575	524	+	hypothetical protein Aphrodite1_0127 [*Vibrio* phage Aphrodite1]	0	99%	AUR80940.1
126	139470	140951	1482	493	+	hypothetical protein Aphrodite1_0126 [*Vibrio* phage Aphrodite1]	0	99%	AUR80947.1
127	141037	142452	1416	471	+	hypothetical protein Aphrodite1_0125 [*Vibrio* phage Aphrodite1]	0	99%	AUR80952.1
128	142519	143145	627	208	+	hypothetical protein Aphrodite1_0124 [*Vibrio* phage Aphrodite1]	3.00E-149	99%	AUR81026.1
129	143198	143518	321	106	-	hypothetical protein Aphrodite1_0123 [*Vibrio* phage Aphrodite1]	8.00E-74	100%	AUR81090.1
130	143614	145716	2103	700	+	hypothetical protein Aphrodite1_0122 [*Vibrio* phage Aphrodite1]	0	99%	AUR80928.1
131	145694	147679	1986	661	+	DNA-directer RNA polymerase beta subunit [*Vibrio* phage Aphrodite1]	0	99%	AUR80932.1
132	147688	149220	1533	510	+	phage terminase, large subunit @ intein-containing [*Vibrio* phage pTD1]	0	93%	BAW98322.1
133	149287	149637	351	116	+	hypothetical protein Aphrodite1_0119 [*Vibrio* phage Aphrodite1]	1.00E-76	98%	AUR81082.1
134	149621	150058	438	145	+	hypothetical protein Aphrodite1_0118 [*Vibrio* phage	5.00E-97	100%	AUR81065.1

						Aphrodite1]			
135	150031	150438	408	135	+	hypothetical protein Aphrodite1_0117 [*Vibrio* phage Aphrodite1]	6.00E-92	99%	AUR81067.1
136	150451	150891	441	146	+	hypothetical protein Aphrodite1_0116 [*Vibrio* phage Aphrodite1]	6.00E-86	95%	AUR81061.1
137	151064	152362	1299	432	-	hypothetical protein Aphrodite1_0115 [*Vibrio* phage Aphrodite1]	0	97%	AUR80959.1
138	152337	153263	927	308	-	putative virion structural protein [*Vibrio* phage Aphrodite1]	0	98%	AUR80990.1
139	153293	154018	726	241	+	hypothetical protein Aphrodite1_0113 [*Vibrio* phage Aphrodite1]	3.00E-171	97%	AUR81011.1
140	154011	155633	1623	540	+	hypothetical protein Aphrodite1_0112 [*Vibrio* phage Aphrodite1]	0	97%	AUR80937.1
141	155702	156088	387	128	+	hypothetical protein Aphrodite1_0111 [*Vibrio* phage Aphrodite1]	2.00E-87	99%	AUR81072.1
142	156144	156722	579	192	+	hypothetical protein Aphrodite1_0110 [*Vibrio* phage Aphrodite1]	1.00E-131	98%	AUR81035.1
143	156874	157233	360	119	+	hypothetical protein Aphrodite1_0109 [*Vibrio* phage Aphrodite1]	1.00E-57	88%	AUR81079.1
144	157296	159062	1767	588	-	putative DNA polymerase [*Vibrio* phage Aphrodite1]	0	99%	AUR80935.1
145	159146	160474	1329	442	+	putative virion structural protein [*Vibrio* phage Aphrodite1]	0	94%	AUR80958.1
146	160476	160886	411	136	+	hypothetical protein Aphrodite1_0106 [*Vibrio* phage Aphrodite1]	2.00E-91	97%	AUR81066.1
147	160924	162360	1437	478	+	tail length tape-measure protein [*Vibrio* phage Aphrodite1]	0	98%	AUR80948.1
148	162357	163079	723	240	+	hypothetical protein Aphrodite1_0104 [*Vibrio* phage Aphrodite1]	3.00E-175	98%	AUR81013.1
149	163126	166065	2940	979	-	hypothetical protein Aphrodite1_0103 [*Vibrio* phage Aphrodite1]	0	99%	AUR80916.1
150	166047	167090	1044	347	-	putative virion structural protein [*Vibrio* phage Aphrodite1]	0	99%	AUR80982.1
151	167126	168247	1122	373	+	capsid and scaffold protein [*Vibrio* phage Aphrodite1]	0	98%	AUR80976.1
152	168247	169128	882	293	+	putative virion structural protein [*Vibrio* phage Aphrodite1]	0	99%	AUR80996.1
153	169140	169649	510	169	+	hypothetical protein Aphrodite1_0099 [*Vibrio* phage	5.00E-122	99%	AUR81041.1

						Aphrodite1]			
154	169642	170907	1266	421	+	hypothetical protein Aphrodite1_0098 [*Vibrio* phage Aphrodite1]	0	98%	AUR80963.1
155	170910	171863	954	317	+	hypothetical protein Aphrodite1_0097 [*Vibrio* phage Aphrodite1]	0	99%	AUR80989.1
156	171921	173204	1284	427	+	hypothetical protein Aphrodite1_0096 [*Vibrio* phage Aphrodite1]	0	98%	AUR80961.1
157	173313	174467	1155	384	+	hypothetical protein Aphrodite1_0095 [*Vibrio* phage Aphrodite1]	0	99%	AUR80969.1
158	174484	175683	1200	399	+	hypothetical protein Aphrodite1_0094 [*Vibrio* phage Aphrodite1]	0	99%	AUR80968.1
159	175788	177038	1251	416	+	hypothetical protein Aphrodite1_0093 [*Vibrio* phage Aphrodite1]	0	98%	AUR80964.1
160	177079	178338	1260	419	+	hypothetical protein Aphrodite1_0092 [*Vibrio* phage Aphrodite1]	5.00E-80	61%	AUR80962.1
161	178483	179721	1239	412	+	hypothetical protein Aphrodite1_0091 [*Vibrio* phage Aphrodite1]	0	99%	AUR80966.1
162	179812	180900	1089	362	+	hypothetical protein Aphrodite1_0090 [*Vibrio* phage Aphrodite1]	0	98%	AUR80979.1
163	180910	182322	1413	470	+	hypothetical protein Aphrodite1_0089 [*Vibrio* phage Aphrodite1]	0	97%	AUR80953.1
164	182392	183996	1605	534	+	putative virion structural protein [*Vibrio* phage Aphrodite1]	0	98%	AUR80939.1
165	184000	185289	1290	429	+	putative virion structural protein [*Vibrio* phage Aphrodite1]	0	99%	AUR80960.1
166	185279	185992	714	237	+	hypothetical protein Aphrodite1_0086 [*Vibrio* phage Aphrodite1]	3.00E-169	98%	AUR81016.1
167	185994	187325	1332	443	+	putative virion structural protein [*Vibrio* phage Aphrodite1]	0	99%	AUR80956.1
168	187445	188857	1413	470	+	hypothetical protein Aphrodite1_0084 [*Vibrio* phage Aphrodite1]	0	73%	AUR80949.1
169	188912	189310	399	132	+	hypothetical protein Aphrodite1_0083 [*Vibrio* phage Aphrodite1]	6.00E-84	91%	AUR81071.1
170	189363	189524	162	53	+	hypothetical protein AMSG_03854 [Thecamonas trahens ATCC 50062]	6	48%	XP_013759757.1

171	189526	189837	312	103	+	hypothetical protein Aphrodite1_0081 [*Vibrio* phage Aphrodite1]	5.00E-62	87%	AUR81091.1
172	190075	190914	840	279	+	hypothetical protein Aphrodite1_0080 [*Vibrio* phage Aphrodite1]	0	90%	AUR81000.1
173	190911	191408	498	165	+	hypothetical protein Aphrodite1_0079 [*Vibrio* phage Aphrodite1]	2.00E-111	92%	AUR81045.1
174	191405	191911	507	168	+	hypothetical protein Aphrodite1_0078 [*Vibrio* phage Aphrodite1]	8.00E-119	98%	AUR81042.1
175	191926	192330	405	134	+	hypothetical protein Aphrodite1_0077 [*Vibrio* phage Aphrodite1]	4.00E-89	96%	AUR81069.1
176	192327	192785	459	152	+	hypothetical protein Aphrodite1_0076 [*Vibrio* phage Aphrodite1]	9.00E-100	94%	AUR81054.1
177	192790	193125	336	111	+	none			
178	193194	195056	1863	620	+	hypothetical protein Aphrodite1_0074 [*Vibrio* phage Aphrodite1]	0	59%	AUR80934.1
179	195058	195408	351	116	+	hypothetical protein Aphrodite1_0073 [*Vibrio* phage Aphrodite1]	3.00E-77	98%	AUR81081.1
180	195470	195796	327	108	+	hypothetical protein Aphrodite1_0072 [*Vibrio* phage Aphrodite1]	4.00E-69	96%	AUR81087.1
181	195866	197404	1539	512	-	DNA helicase [*Vibrio* phage Aphrodite1]	0	100%	AUR80942.1
182	197450	198016	567	188	+	hypothetical protein Aphrodite1_0070 [*Vibrio* phage Aphrodite1]	2.00E-136	100%	AUR81033.1
183	198116	200311	2196	731	+	capsid and scaffold protein [*Vibrio* phage Aphrodite1]	0	99%	AUR80923.1
184	200495	201565	1071	356	+	hypothetical protein Aphrodite1_0068 [*Vibrio* phage Aphrodite1]	0	99%	AUR80980.1
185	201609	203282	1674	557	+	hypothetical protein Aphrodite1_0067 [*Vibrio* phage Aphrodite1]	0	100%	AUR80936.1
186	203279	205762	2484	827	+	hypothetical protein Aphrodite1_0066 [*Vibrio* phage Aphrodite1]	0	98%	AUR80920.1
187	205822	206385	564	178	+	hypothetical protein Aphrodite1_0065 [*Vibrio* phage Aphrodite1]	4.00E-125	93%	AUR81034.1
188	206389	207393	1005	334	+	hypothetical protein Aphrodite1_0064 [*Vibrio* phage	0	99%	AUR80985.1

						Aphrodite1]			
189	207390	208139	750	249	+	hypothetical protein Aphrodite1_0063 [*Vibrio* phage Aphrodite1]	3.00E-180	98%	AUR81008.1
190	208290	208733	444	147	+	hypothetical protein Aphrodite1_0062 [*Vibrio* phage Aphrodite1]	9.00E-101	100%	AUR81059.1
191	208818	209183	366	121	+	hypothetical protein Aphrodite1_0061 [*Vibrio* phage Aphrodite1]	1.00E-84	99%	AUR81078.1
192	209234	212131	2898	965	-	hypothetical protein Aphrodite1_0060 [*Vibrio* phage Aphrodite1]	0	97%	AUR80917.1
193	212152	214263	2112	703	+	hypothetical protein Aphrodite1_0059 [*Vibrio* phage Aphrodite1]	0	99%	AUR80927.1
194	214322	215236	915	304	+	putative virion structural protein [*Vibrio* phage Aphrodite1]	0	99%	AUR80991.1
195	215236	215838	603	200	+	hypothetical protein Aphrodite1_0057 [*Vibrio* phage Aphrodite1]	3.00E-128	99%	AUR81036.1
196	215819	216172	354	117	+	hypothetical protein Aphrodite1_0056 [*Vibrio* phage Aphrodite1]	7.00E-81	99%	AUR81080.1
197	216220	216660	441	146	-	hypothetical protein [*Vibrio* phage pTD1]	3.00E-42	61%	BAW98259.1
198	216662	217051	390	129	-	hypothetical protein Aphrodite1_0054 [*Vibrio* phage Aphrodite1]	1.00E-87	98%	AUR81074.1
199	217055	218290	1236	411	-	hypothetical protein Aphrodite1_0053 [*Vibrio* phage Aphrodite1]	0	99%	AUR80967.1
200	218369	220510	2142	713	+	tail fiber protein [*Vibrio* phage Aphrodite1]	0	96%	AUR80925.1
201	220631	223900	3270	1089	+	minor tail protein [*Vibrio* phage Aphrodite1]	0	96%	AUR80914.1
202	223939	226149	2211	736	+	minor tail protein [*Vibrio* phage Aphrodite1]	0	98%	AUR80922.1
203	226151	228661	2511	836	+	hypothetical protein Aphrodite1_0049 [*Vibrio* phage Aphrodite1]	0	96%	AUR80919.1
204	228710	229762	1053	350	-	phage baseplate protein [*Vibrio* phage Aphrodite1]	0	99%	AUR80981.1
205	229817	232294	2478	825	+	DNA double-strand break repair Rad50 ATPase [*Vibrio* phage Aphrodite1]	0	99%	AUR80921.1
206	232385	232657	273	90	+	hypothetical protein Aphrodite1_0046 [*Vibrio* phage Aphrodite1]	3.00E-60	100%	AUR81096.1

207	232718	233161	444	147	+	hypothetical protein Aphrodite1_0045 [*Vibrio* phage Aphrodite1]	9.00E-105	100%	AUR81058.1
208	233171	233806	636	211	+	hypothetical protein Aphrodite1_0044 [*Vibrio* phage Aphrodite1]	2.00E-155	99%	AUR81024.1
209	233793	235754	1962	653	+	DNA ligase [*Vibrio* phage Aphrodite1]	0	96%	AUR80933.1
210	235785	236087	303	100	+	hypothetical protein Aphrodite1_0042 [*Vibrio* phage Aphrodite1]	4.00E-63	95%	AUR81092.1
211	236116	237360	1245	414	+	hypothetical protein Aphrodite1_0041 [*Vibrio* phage Aphrodite1]	0	98%	AUR80965.1
212	237418	237900	483	160	+	hypothetical protein Aphrodite1_0040 [*Vibrio* phage Aphrodite1]	9.00E-114	99%	AUR81048.1
